# Realization of Dirac Cones in Few Bilayer Sb(111) Films by Surface Modification

**DOI:** 10.1186/s11671-015-1043-8

**Published:** 2015-08-21

**Authors:** Hui Pan, Xue-Sen Wang

**Affiliations:** Institute of Applied Physics and Materials Engineering, Faculty of Science and Technology, University of Macau, Macao, SAR China; Department of Physics, National University of Singapore, 2 Science Drive 3, Singapore, 117542 Singapore

**Keywords:** Few bilayer Sb(111) films, Dirac cones, Surface modification, First-principles calculations

## Abstract

We report the first-principle study on the recovery and linearization of Dirac cones in the electronic band structures of a few bilayer Sb(111) films (*n*-BL Sb) by surface modification. Due to the interaction between the surface states on the two surfaces of a free-standing film, the distorted Dirac cone in *n*-BL Sb(111) (*n* < 5) disappears. We demonstrate that the Dirac cone can be restored by functionalizing one surface with certain atoms including H, Ag, and Au, to reduce the inter-surface interaction. We further show that an ideal Dirac cone with linear dispersion of topological surface states near the zone center can be realized by functionalizing both surfaces of the film with oxygen, which enhances spin-orbital coupling. The realization of Dirac cone by surface functionalization shows promise for applications of topologic materials to spintronic devices and their operation in complicated conditions.

**Electronic supplementary material:**

The online version of this article (doi:10.1186/s11671-015-1043-8) contains supplementary material, which is available to authorized users.

## Background

In solid-state materials, the modification of electronic states (including spin states) at the interface and on the surface can strongly affect the electronic and magnetic properties of materials. By engineering the interfaces and surfaces of materials based on such modification, devices with versatile functions can be realized [1]. In recent years, a new class of materials, topological insulators (TIs) [2–15], has attracted extensive attention in condensed-matter physics and materials science. TIs in two or three dimensions (2D or 3D) have a nontrivial band order and a bandgap, often generated by the spin-orbit coupling (SOC) effect [16–18]. The boundaries (surfaces and interfaces in 3D or edges in 2D) of a TI have gapless states that are protected by time-reversal symmetry. The boundary states of a TI lead to the formation of robust conducting channels with properties that are distinguished from any other low-dimensional systems [18]. These states are predicted to have special properties, such as supporting dissipationless spin currents that may have potential applications in spintronics and quantum computation. The topological surface states can also play a vital role in facilitating surface reactions by serving as an effective electron bath, which may provide new design of heterogeneous catalysts [19]. TIs have also shown promise for thermoelectric applications [20].

The TIs discovered so far include simple semimetals (Sb and Bi) [4, 21–29], alloys (Bi_1 − *x*_Sb_*x*_) [4, 16], binary compounds (HgTe, Bi_2_Se_3_, Sb_2_Te_3_, and Bi_2_Te_3_) [4–10, 20, 30–36], and ternary semiconducting Heusler compounds [12–14]. As a simple elemental TI, semimetal Sb has the same nontrivial topological order as Bi_1 − *x*_Sb_*x*_ for *x* > 0.07 [4, 21]. Two Rashba-type spin-split surface bands on Sb(111), connected to the bulk conduction and the valence bands separately, result in a single Dirac cone within the bulk bandgap of Sb [21–29]. Importantly, Sb(111) thin films with a thickness of ≤15 bilayers (BL; with 1 BL = 3.75 Å) can be converted into a 3D TI due to the quantum confinement effect which opens up a positive bandgap for the bulk states [24–26]. Such Sb thin films can be obtained by Sb deposition on Si(111) and other substrates, and the electronic states in *n*-BL Sb(111) thin films with *n* ≥ 4 have been studied using angle-resolved photoelectron spectroscopy and scanning tunneling spectroscopy (STS) [24, 26], which provides direct experimental evidences for the theoretical predications. To date, however, the topological properties of *n*-BL Sb(111) ultrathin films with *n* < 4 have not been studied experimentally. On the other hand, the inter-surface coupling of the surface states opens up a gap at the surface Brillouin zone (SBZ) center $\overline{\varGamma},$ destroying the Dirac cone [24–26]. In this work, we investigate the effects of thickness on the electronic properties of Sb thin films using the first-principles calculations. We find that, although the Dirac cone disappears for *n*-BL Sb(111) ultrathin films with *n* < 5, the Dirac cone can be recovered by surface modification. We predict that the surface modification provides an alternative way for the formation of topological ordering.

## Methods

The first-principles calculations were carried out based on the density function theory (DFT) [37] and the Perdew-Burke-Ernzerhof generalized gradient approximation (PBE-GGA) [38]. The projector augmented wave (PAW) scheme is incorporated in the Vienna *ab initio* simulation package (VASP) [39, 40]. The Monkhorst and Pack scheme of *k*-point sampling is used for integration over the first Brillouin zone [41]. A 9 × 9 × 1 grid for *k*-point sampling and an energy cutoff of 400 eV are used for geometry optimization. Excellent convergence is obtained using these parameters, and the total energy is converged to 2.0 × 10^−5^ eV/atom. A large supercell dimension with a vacuum of 25 Å separating the Sb(111) thin films is used to avoid any interaction between the neighboring slabs.

## Results and discussion

**Fig. 1:**
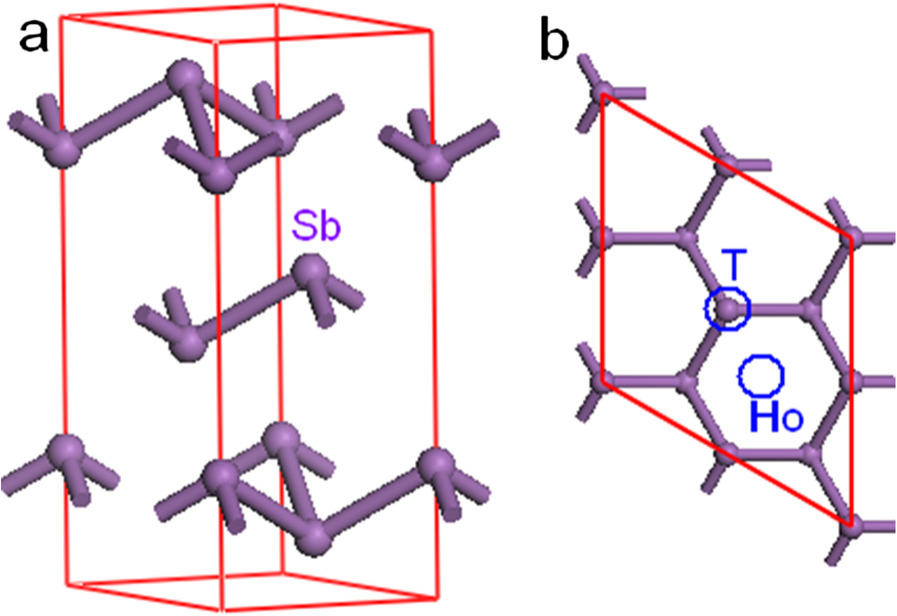
**a** Crystal Structure of Sb and **b** top view of one bilayer Sb in [111] direction. *T* top position, *H*_*O*_ hollow site

Sb has the rhombohedral A7 crystal structure with space group $R\overline{3}m$ (Fig. 1). To obtain the lattice parameters, the Sb bulk was first optimized. The optimized lattice parameters are *a* = 4.398 Ǻ, *c* = 11.369 Ǻ, and *d* = 2.283 Ǻ (the distance between two bilayers along Z direction. If *d* is defined as the distance along Z direction between two atoms in the same bilayers, then *d* = 1.507 Ǻ), in good agreement with the experimental values [42].

**Fig. 2:**
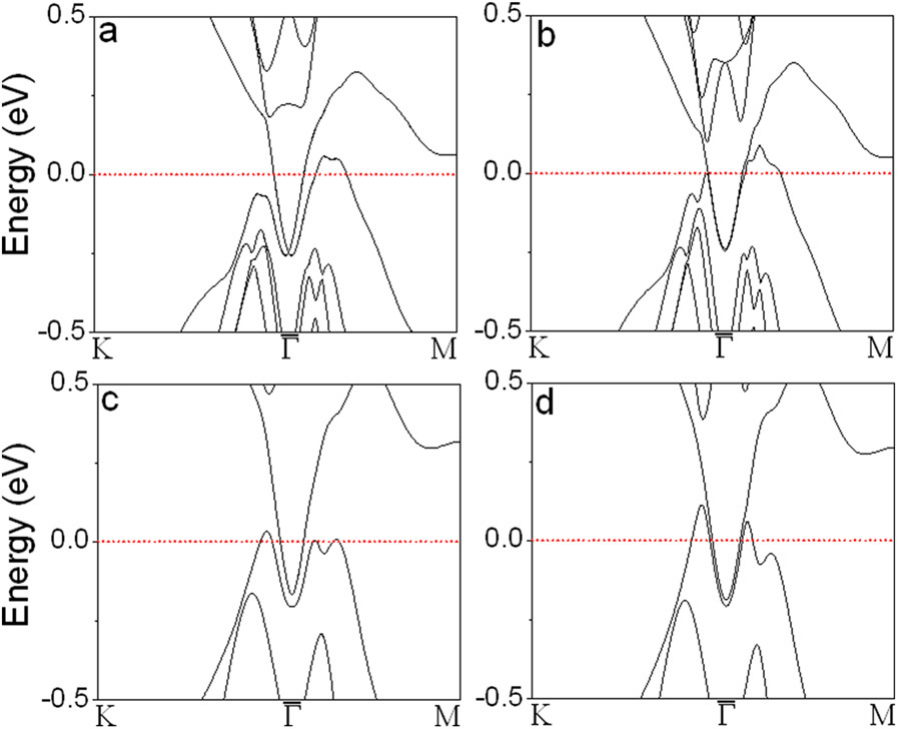
Calculated band structures of Sb(111) thin films: **a** 6 BL with SOC included in the calculations, **b** 6 BL without SOC, **c** 3 BL with SOC, and **d** 3 BL without SOC

Sb thin films with the thickness varying from 1 to 10 BL oriented in [111] direction are constructed using these parameters first. Next, the geometries are fully optimized for each thickness. The lattice constant of 1-BL Sb(111) is contracted to *a* = 4.124 Ǻ. For the 2-BL film, we get *a* = 4.260 Ǻ and *d* = 2.488 Ǻ. From *n* = 3 onwards, the lattice parameters of the *n*-BL films are almost equal to the bulk values. The calculated electronic structures of Sb thin films are shown in Fig. 2 and Additional file 1: Figure S1. For *n*-BL Sb(111) films with *n* > 5, the two surface bands obtained from calculations with SOC included are degenerate at the $\overline{\varGamma}$ point and separated elsewhere in the Brillion zone (Fig. 2a), because of the Rashba effect [23]. By switching off the SOC, the Dirac cone is not observable and the surface bands are degenerate within the gap (Fig. 2b), indicating the system topologically trivial. The Dirac cones on the *n*-BL Sb(111) for *n* > 4 are distorted from the ideally linear dispersion near the $\overline{\varGamma}$ because of the relatively weak SOC of Sb [43]. In the cases of *n* = 5 and *n* = 4, the band structures with SOC show that the degeneracy at $\overline{\varGamma}$ is lifted, but the surface states keep degenerate within the bandgaps of the bulk bands if without SOC (Additional file 1: Figure S1). With further reducing the thickness, the degeneracy at $\overline{\varGamma}$ is not observable even without SOC included in the calculations (Fig. 2c, d), because of the significant coupling between the states on two surfaces in the free-standing thin films of *n* < 5. To recover the Dirac cones in *n*-BL Sb(111) for *n* = 1–4, we tried to reduce the coupling by modifying the film surface. In the following, we investigate *n*-BL Sb(111) for *n* = 1–10 with one surface covered by a layer of atoms, including H, O, Ag, and Au.

**Fig. 3:**
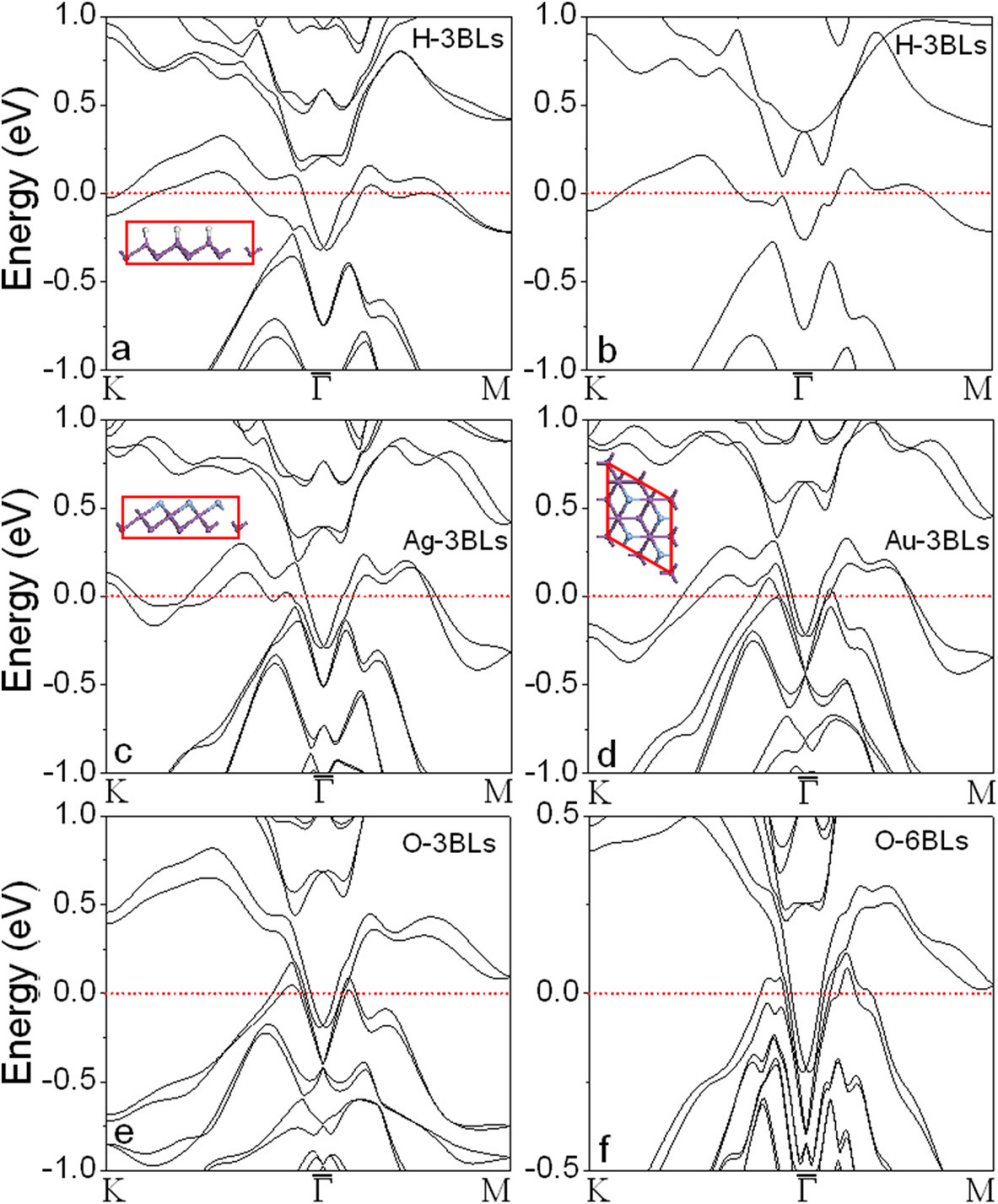
Calculated band structures of Sb(111) films with surface coating: **a** 3 BL with one surface covered by H atoms (H-3 BLs), **b** H-3 BL without SOC, **c** Ag-3 BL, **d** Au-3 BL, **e** O-3 BL, and **f** O-6 BL

The stable sites for the adsorption of the atoms are determined first. We find that the H and O atoms prefer the top positions (T in Fig. 1b) above Sb atoms at the surface, while the hollow sites (H_O_ in Fig. 1b) are stable positions to hold the Ag and Au atoms (see the insets in Fig. 3). The lateral period *a* is extended to 4.858 and 4.650 Ǻ for of H-covered 1- and 2-BL Sb(111), respectively. In contrast, the lateral period is contracted to 4.156 and 4.266 Ǻ for the Ag-covered 1- and 2-BL samples, respectively, and is contracted further (4.105 and 4.25 Ǻ) in Au-covered cases. The lattice relaxation is <1 % for Sb *n*-BLs (*n* < 3) with one surface covered by O. The lattice parameter is gradually restored to the bulk value with increasing film thickness. The calculated Sb–H, Sb–O, Sb–Ag, and Sb–Au bond lengths are 1.782, 1.837, 2.817, and 2.78 Ǻ, respectively. The calculated formation energies are 1.2, −0.63, 0.87, and 0.44 eV for H, O, Ag, and Au atoms on Sb n-BLs (*n* > 2), respectively. The negative formation energy of O indicates that the process is exothermic and can be easily realized in experiments.

**Fig. 4:**
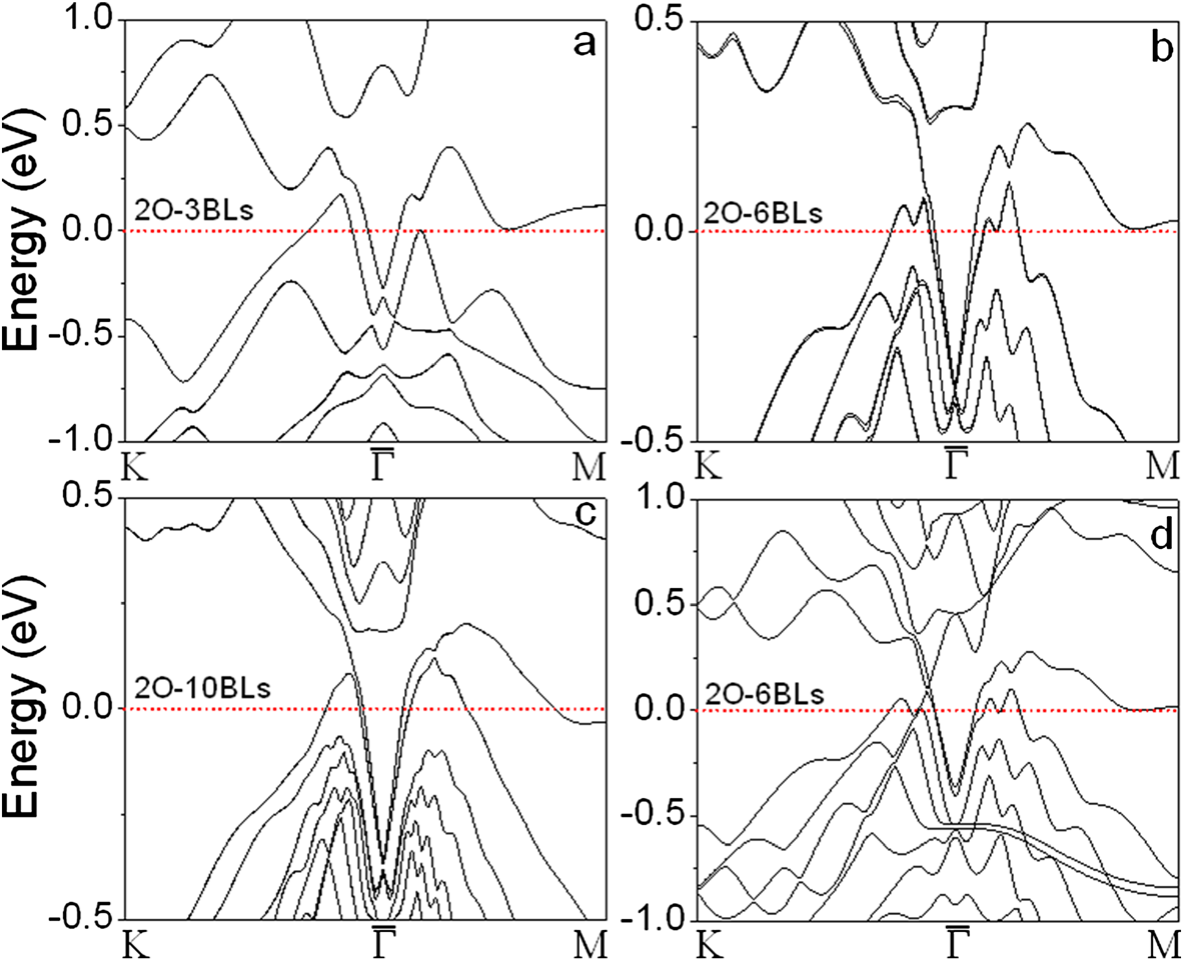
Calculated band structures of Sb(111) films with O atoms adsorbed on both surfaces: **a** 3 BL, **b** 6 BL, and **c** 10 BL with SOC included in the calculations; **d** 6 BL without SOC

The electronic structures of the A-covered (A═H, O, Ag, and Au) films clearly show that the Dirac cones at the $\overline{\varGamma}$ points in the *n*-BLs (*n* < 5) are recovered as shown in Fig. 3 and supporting information (Additional file 1: Figure S2 and S3). By comparing the band structures of H-covered 3 BLs calculated with (Fig. 3a) and without SOC (Fig. 3b), we note that the topological surface states are attributed to the SOC. The topological surface states near the $\overline{\varGamma}$ point are almost equal to those in *n*-BLs (*n* > 5) because the adsorbates remove the coupling between the states on the two surfaces by forming sp hybridization between Sb and adsorbed atoms (H, O, Ag, and Au) on the covered surface. Interestingly, we can see the two Dirac cones on O-covered Sb films (Fig. 3e, f): the distorted one is related to the uncovered Sb(111) surface (just below the Fermi level), and the perfect linear Dirac cone (below the distorted Dirac cone) is attributed to the oxidized surface and not affected by increasing the thickness (Fig. 3e, f). The linear Dirac bands are further confirmed by covering both surfaces of Sb film with O atoms (Fig. 4a–c and Additional file 1: Figure S4). We see that the Dirac cones keep linear near the surface zone center. The Dirac cone cannot be observed from the band structure calculated without SOC (see Fig. 4d), elucidating that the interface bands (Sb–O) are originated from the SOC. It had been reported that single-sheet-coated Sb films can form topological surface and interface states by preserving time-reversal symmetry [29]. The linearized Dirac bands in O-covered Sb films are contributed to time-reversal symmetry and reduced surface-surface (or interface-interface) interaction. We also notice that there is a gap at the Dirac point on O-covered 3- and 10-BLs Sb films (Fig. 4a, c). The Dirac cones may be closed or opened depending on the thickness because of the combined effect of SOC and surface-surface interaction. The linear dispersion of bands near the $\overline{\varGamma}$ point in the Au-covered 3 BLs (Au-3 BLs, Fig. 3d) is not a Dirac cone because all of the bands are connected to the bulk valence band. The Dirac-like shape disappears by increasing the thickness (*n* > 4) and covering both surfaces of Sb films with Au atoms (Additional file 1: Figure S5).

**Fig. 5:**
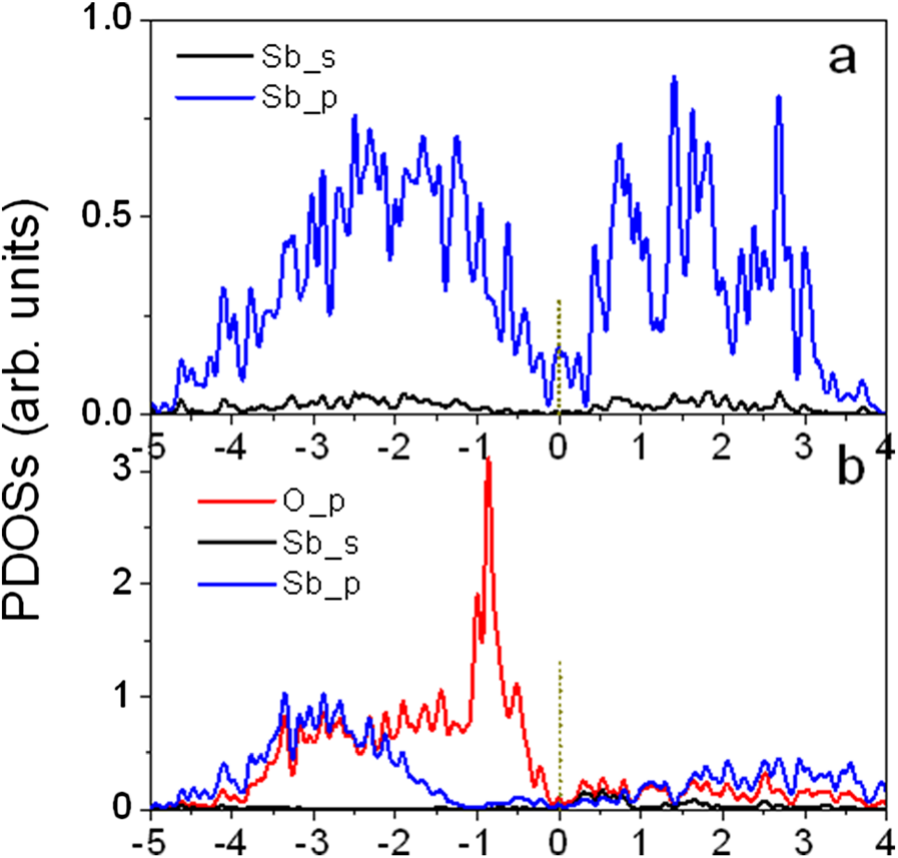
Calculated partial density of states (PDOSs) of 6-BL Sb(111): **a** without coating and **b** with O atoms adsorbed on both surfaces

The calculated partial density of states (PDOSs) further reveals the origin of the linear dispersions of the interface states on O-covered Sb films (see Fig. 5). For Sb films without O-covering, the surface states are totally attributed to the Sb-*p* electrons (Fig. 5a). The main part of the Sb-*p* electrons in the valence band is within the energy range from −3 to −1 eV under the Fermi level. In the O-covered Sb films, the Sb-*p* electrons are highly hybridized with O-*p* electrons in the range of −4 to −2 eV. The O-*p* electrons are dominant around −0.9 eV (see Fig. 5b), contributing to the interface states. The energy states around −0.5 and −0.25 eV are attributed to the partial hybridization between the O-*p* and Sb-*p* electrons. Clearly, the surface states are enhanced by the O-covering, leading to the increase of carrier density near the Fermi level.

## Conclusions

In summary, we predict that the topological surface/interface states of Sb thin film with a few bilayers can be recovered and modified by surface coating. We show that the ideal Dirac cone is realized by covering the two surfaces of a free-standing Sb film with oxygen atoms because of the enhanced SOC at the O-Sb interfaces. The present work on engineering the topological Dirac cone by surface coating provides an alternative and simple way to utilize topological materials for their applications in nanodevices.

## Additional file

Additional file 1: Figure S1. Calculated band structure of a few bilayer (BL) Sb films with and without SOC. (a) 1 BL, (c) 2 BL, (e) 4 BL, (g) 5 BL, and (i) 10 BL with SOC; (b) 1 BL, (d) 2 BL, (f) 4 BL, (h) 5 BL, and (j) 10 BL without SOC. **Figure S2.** Calculated band structures of H-covered a few bilayers (BL) Sb films with SOC: (a) 1 BL, (b) 2 BL, (c) 4 BL, (d) 5 BL, (e) 6 BL, and (f) 10 BL. **Figure S3.** Calculated band structures of Ag-covered a few bilayers (BL) Sb films with SOC: (a) 1 BL, (b) 2 BL, (c) 4 BL, (d) 5 BL, (e) 6 BL, and (f) 10 BL. **Figure S4.** Calculated band structures of a few BL Sb films with two surface covered by O atoms by considering SOC: (a) 4 BL and (b) 5 BL. **Figure S5.** Calculated band structures of 3-BL Sb films with two surface covered by Au atoms by considering SOC. (DOC 131 kb)
