# Supplementary material for: Realization of Dirac Cones in Few Bilayer Sb(111) Films by Surface Modification
Source: Nanoscale Res Lett. 2015 Aug 21;10:334. doi: 10.1186/s11671-015-1043-8 (PMC4545755; doi:10.1186/s11671-015-1043-8)
Supplement: Additional file 1: Figure S1. — Calculated band structure of a few bilayer (BL) Sb films with and without SOC. (a) 1 BL, (c) 2 BL, (e) 4 BL, (g) 5 BL, and (i) 10 BL with SOC; (b) 1 BL, (d) 2 BL, (f) 4 BL, (h) 5 BL, and (j) 10 BL without SOC. Figure S2. Calculated band structures of H-covered a few bilayers (BL) Sb films with SOC: (a) 1 BL, (b) 2 BL, (c) 4 BL, (d) 5 BL, (e) 6 BL, and (f) 10 BL. Figure S3. Calculated band structures of Ag-covered a few bilayers (BL) Sb films with SOC: (a) 1 BL, (b) 2 BL, (c) 4 BL, (d) 5 BL, (e) 6 BL, and (f) 10 BL. Figure S4. Calculated band structures of a few BL Sb films with two surface covered by O atoms by considering SOC: (a) 4 BL and (b) 5 BL. Figure S5. Calculated band structures of 3-BL Sb films with two surface covered by Au atoms by considering SOC. (DOC 131 kb) [file 11671_2015_1043_MOESM1_ESM.doc]

**Supplementary Information to “Realization of Dirac Cones in Few-Bilayers Sb(111) Films by Surface Modification”**

Hui Pan and Xue-Sen Wang


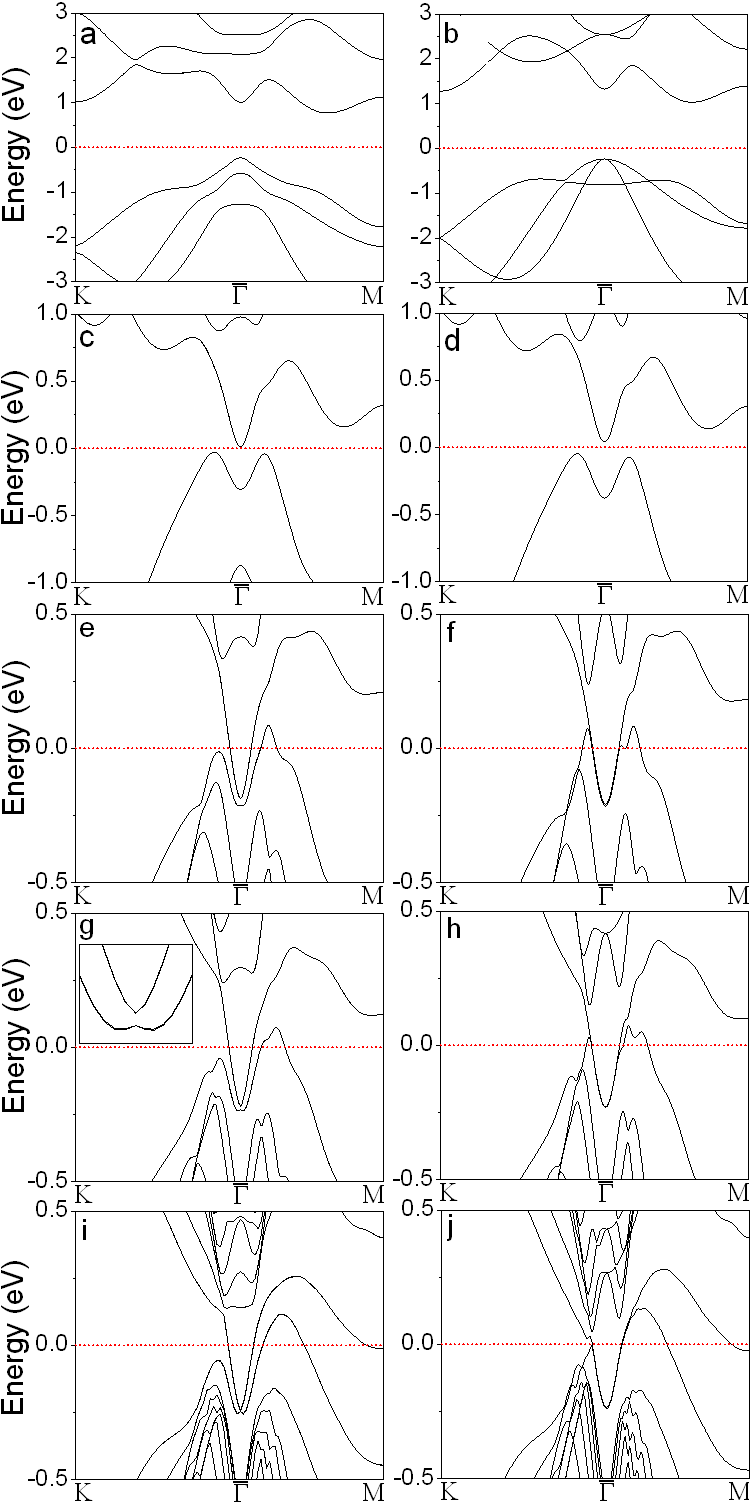


Figure S1, Calculated band structure of a few bilayers (BL) Sb films with and without SOC. (a) 1BL, (c) 2BL, (e) 4BL, (g) 5BL, and (i) 10BL with SOC; (b) 1BL, (d) 2BL, (f) 4BL, (h) 5BL, and (j) 10BL without SOC


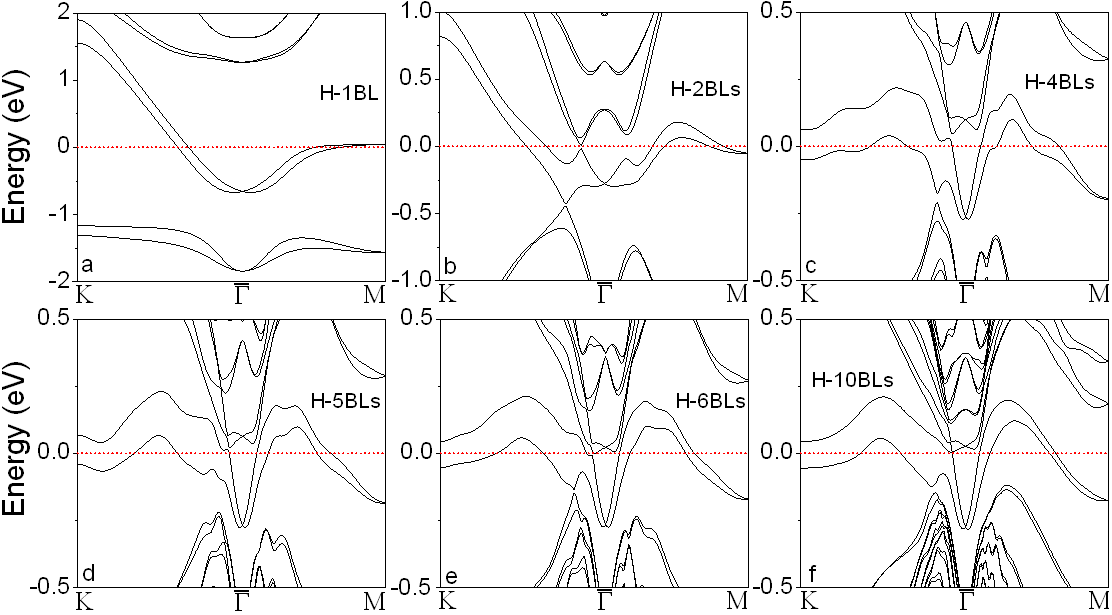


Figure S2, Calculated band structures of H-covered a few bilayers (BL) Sb films with SOC: (a) 1BL, (b) 2BL, (c) 4BL, (d) 5BL, (e) 6BL, and (f) 10 BL.


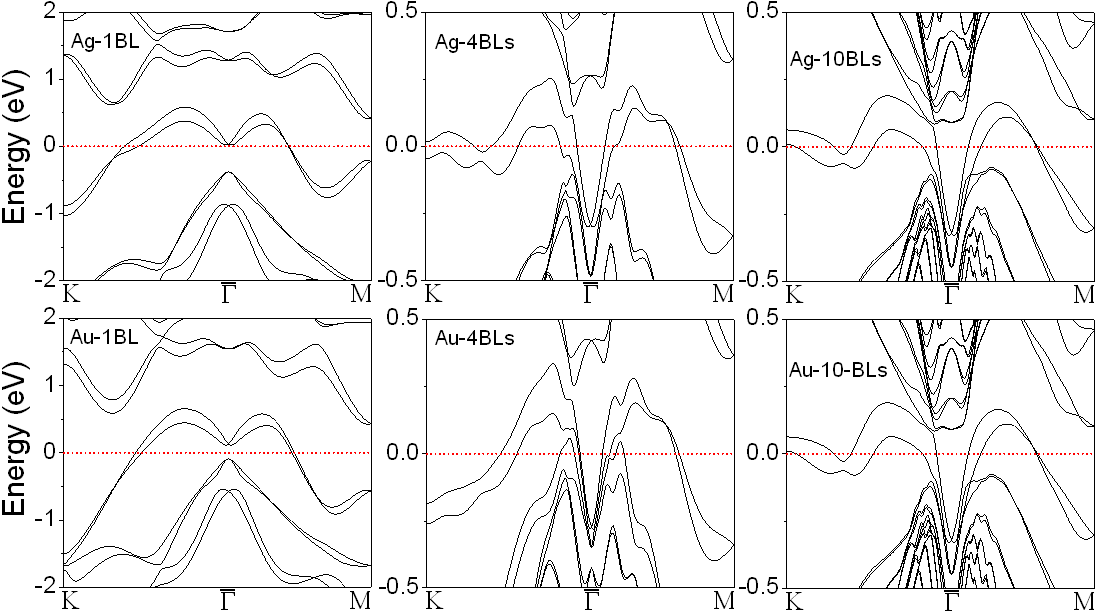


Figure S3, Calculated band structures of Ag-covered a few bilayers (BL) Sb films with SOC: (a) 1BL, (b) 2BL, (c) 4BL, (d) 5BL, (e) 6BL, and (f) 10 BL.


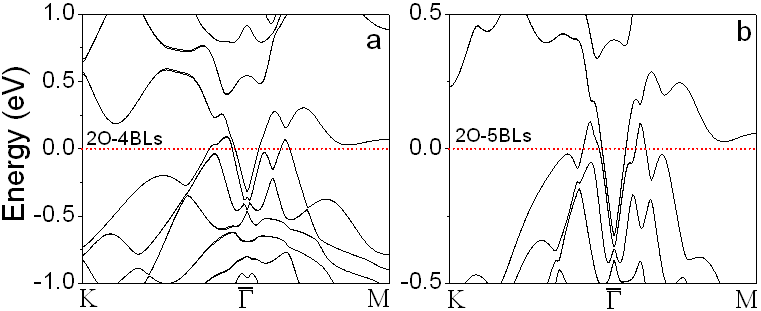


Figure S4, Calculated band structures of a few BL Sb films with two surface covered by O atoms by considering SOC: (a) 4BL and (b) 5BL.


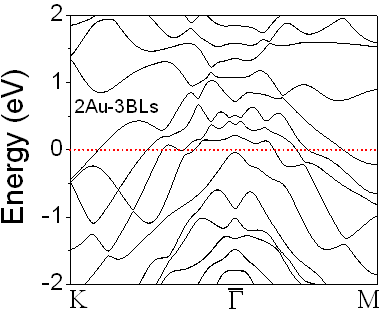


Figure S5, Calculated band structures of 3BL Sb films with two surface covered by Au atoms by considering SOC.
